# Knowledge on mother-to-child transmission of HIV, and sexuality and fertility desires among people living with HIV in North-Central, Nigeria

**DOI:** 10.11604/pamj.2021.40.64.31455

**Published:** 2021-09-28

**Authors:** Chikwendu Amaike, Tolulope Olumide Afolaranmi, Blessing Adaku Amaike, Hadiza Agbo, Olumide Abiodun

**Affiliations:** 1 Department of Community Medicine, College of Health and Medical Sciences, Babcock University, Ilishan Remo, Ogun State, Nigeria,; 2 Seventh-Day Adventist Hospital, Jengre, Plateau State, Nigeria,; 3 Department of Community Medicine, University of Jos and Jos University Teaching Hospital, Jos, Plateau State, Nigeria,; 4 Department of Pharmaceutical Technology, Faculty of Pharmaceutical Sciences, University of Jos, Plateau State, Nigeria

**Keywords:** Mother-to-child transmission, sexuality, fertility, Nigeria, HIV, reproductive health services, anti-retroviral treatment

## Abstract

**Introduction:**

mother-to-child transmission (MTCT) is the transmission of HIV from a mother to the child during pregnancy, labour and breastfeeding. People living with HIV (PLHIV) are sexually active and also HIV can be transmitted while trying to achieve pregnancy involving unprotected hetero-sexual intercourse. Fertility desire among PLHIV is increasing due to improved quality of life and survival following commencement of anti-retroviral treatment and available reproductive health services. The objective of the study was to determine the association between knowledge on MTCT of HIV and sexuality and fertility desire.

**Methods:**

this study was descriptive cross-sectional applying systematic sampling technique among PLHIV using semi-structured interviewer administered questionnaires. Data was analysed using SPSS version 23.0. Chi square test was used for statistical analysis. At 95% confidence interval a P-value of <0.05 was considered to be statistically significant.

**Results:**

a total of 168 PLHIV were studied, 63.3% females and 36.7% males. Majority (81.5%) of the respondents were sexually active and 64.1% had fertility desire. On awareness of MTCT 62.5% had heard of MTCT but only 28.2% had good knowledge. No association was found between knowledge of MTCT and sexuality and fertility desire respectively.

**Conclusion:**

PLHIV had high awareness but poor knowledge on MTCT of HIV, hence the need for healthcare workers to provide sexual and reproductive health counselling including information on MTCT to both male and female PLHIV during routine clinic visits.

## Introduction

Human immune deficiency virus (HIV) infection remains a major global public health issue, with about 74.9 million people infected and more than 32 million deaths since the onset of the epidemics up to 2018 [1], and Nigeria having the second largest HIV epidemic in the world and one of the highest rates of new HIV infections in sub-Saharan Africa [2]. The HIV infected mother can transmit the virus via both the horizontal and vertical routes. In the vertical route, a pregnant woman transmits HIV to her unborn child during pregnancy, delivery and breastfeeding. This is referred to as mother-to-child transmission (MTCT) [3,4]. Mother-to-child transmission of HIV, however, be effectively reduced or prevented if both mother and baby are placed on antiretrovirals (ARV). In the absence of ARVs, the rate of HIV infection from MTCT is between 15 to 45% [3]. The comprehensive approach to prevention of mother-to-child transmission (PMTCT) programmes include prevention of new HIV infections among women of childbearing age, prevention of unintended pregnancies among women living with HIV, prevention of HIV transmission from a woman living with HIV to her baby and provision of appropriate treatment, care and support to mothers living with HIV, their children and families [5].

Nigeria accounts for about 30% of all cases of MTCT of HIV globally, but only about 40% of pregnant women in Nigeria living with HIV were on antiretroviral therapy (ART) for PMTCT in 2018 [3]. PLHIV have a right to a satisfying, safe and healthy sexuality and reproductive health, but HIV can be transmitted in the attempt to achieve pregnancy involving unprotected hetero-sexual intercourse. Furthermore, fertility desire among persons living with HIV (PLHIV) is increasing due to improved quality of life and survival following commencement of ARV treatment and also the availability of reproductive health services. Knowledge on MTCT and the utilization of PMTCT services by men and women infected with HIV who are sexually active and have fertility desires may affect the risk of MTCT of HIV. However, little is known about the relationship between the knowledge of MTCT of HIV and the sexuality and fertility desires of PLHIV.

**Study objectives:** this study was aimed at assessing the sexuality, fertility desire and knowledge of MTCT and also to determine the relationship between the knowledge of MTCT and their sexuality and fertility desire.

## Methods

**Study design:**it was a cross-sectional study on knowledge of the clients on MTCT and to relate their knowledge with their sexuality and fertility desire.

**Study location:** this research was done at the Seventh-day Adventist (SDA) Hospital, Jengre, in Bassa Local Government Area (LGA) of Plateau state, Northcentral Nigeria. The hospital is a 58-bed spaced; faith-based secondary health facility established in 1947 [6], which provides comprehensive HIV/AIDS care with support from the AIDS Prevention Initiative Nigeria (APIN) Public Health Initiative. Services provided include HIV testing and counselling services (HTS), ARV drugs, PMTCT, screening and treatment of other sexually transmitted infections (STIs), prophylaxis and treatment of opportunistic infections (OIs), post exposure prophylaxis (PEP), pre-exposure prophylaxis (PreP) and family planning commodities. Adults PLHIV who were on ART at the time of this study were 1,432 and 1,146 of them had been on ART for six months and above, a period of time expected for that viral suppression is expected to have occurred. The HIV programme is integrated into the routine daily out-patient care of the hospital. Patients visit the hospital at least twice a year to pick up their drugs, get clinical assessment and run laboratory tests. Bassa LGA is one of the 17 LGAs in Plateau State and it is located at the northern part of the state, with an area of 1,743 Km^2^and a population of about 200,000 [7]. Plateau state is the twelfth largest state in Nigeria with a population of about 3.5 million people with an area of 30,913km^2^ [8].

**Study population:** this study was conducted among clients who access care for HIV in SDA hospital.

**Eligibility criteria:** males and females 18 years and above who have been on ARVs for a period of six months and above prior to the study were included. Females who were not within the reproductive age group (15-49 years) were however, excluded from the study.

**Study size:** appropriate sample size formula was used to determine the minimum study size [9], where Zα is the value of alpha error at 95% confidence level given as 1.96, P is knowledge of MTCT of HIV found to be 50.3% in Tehran [10] and d is the precision which was set at 15% of 50.3%. A 5% possible non-response was considered and a minimum study size of 168 was gotten.

**Selection of participants:** systematic sampling technique was used to select the study participants. A sampling frame generated from the electronic data base of patients who access HIV care in the hospital showed a total of 1,146 eligible participate for this study. A sampling interval of 7 was gotten by dividing the number of eligible participants by the minimum study size. The first participant was then selected by simple random sampling technique by balloting between the first and second numbers on the sampling frame. The sampling interval of 7 was then applied to select the subsequent participants till the minimum study size was attained.

**Variables:** knowledge on MTCT is the explanatory variable while fertility desire and sexuality are outcome variables.

**Collection of data:** advocacy visits were paid to the administrative committee of SDA hospital and also the leaders of the support group of PLHIV for the hospital to brief them on the aim of the study and solicit their support in carrying out the study. One-day training was conducted for research assistants (two doctors and three nurses) who work in the hospital´s ART clinic for the purpose of administering the questionnaires. Questionnaire adapted from previous studies [11,12] was pre-tested among HIV clients in a comprehensive HIV care site in a border state. This was to correct for any ambiguity and for assessment of face validity. The HIV clinic appointment register was used to determine the routine visit days for the participants. Each participant was reassured of confidentiality, written consents obtained and questionnaire administered after the respondents had been assessed clinically and picked their drugs. This was to ensure that their due benefits were not denied them.

**Grading of responses:** current viral load results were abstracted from each patient´s hospital record and was considered as suppressed if <1000 copies of HIV ribonucleic acid (RNA)/millilitre of plasma or unsuppressed if >1000 copies of HIV RNA/millilitre of plasma [4]. Perceived health status was assessed by asking the question “how is your health in general?” very good was scored 3, good was scored 2 and fair was scored 1 [13]. Sexuality was analysed as sexual behaviour which was categorized as risky and non-risky sexual behaviour [14].

Operational definition for risky sexual behaviours- risky sexual behaviours are activities that will increase the probability that a person engaged in sexual activity with a partner who is infected with a sexually transmitted infection will be infected by the partner. In this study, risky sexual behaviours for HIV infection included use of substance before sexual activity, engaging in sexual activity without use of condom and having sex with more than one partner. A sexual behaviour was considered to be risky if the respondent had sex in the last 12 months but did not use condom or had multiple sexual partners or used substance before sex and non-risky if the respondent did not have sex in the last 12 months or the respondent had sex in the last 12 months but used condom, had only one sexual partner and did not use substance during sex [15-18]. A respondent was considered to have fertility desire if respondent intended to have a child or children in future and those who did not intend to have a child or more children in future were considered as not having fertility desires [19].

Knowledge of MTCT of HIV was assessed using six questions [20,21]. Each correct response was scored 2 and each incorrect response was scored zero. Respondents who had not heard of MTCT of HIV were scored 1 and same for respondents who did not know that MTCT of HIV could be prevented. A maximum attainable score was 12 while the minimum was 2. Over all knowledge was graded as good or poor. A score of 0-5 was considered poor knowledge while a score of ≥6 was considered as good knowledge.

**Data processing and analysis:** the statistical package IBM SPSS version 23.0 was used for data entry and analysis. Sociodemographic and medical related characteristics were presented in frequency tables and expressed as frequencies and percentages. Quantitative variable like age of the respondent was summarised using mean and standard deviation while others like duration of marriage, number of children, duration since knowing HIV status, duration on ARV drugs were grouped for the purpose of analysis. Knowledge on MTCT of HIV which is the explanatory variable was expressed using frequency table as frequencies and percentages. Also, the outcome variables fertility desire and sexuality were presented on frequency table as frequencies and percentages. Chi square test was done to determine the relationship between knowledge on MTCT and sexuality and also knowledge on MTCT and fertility desires of the respondents. Using a confidence interval of 95%, a p value <0.05 was considered to be statistically significant.

**Ethical consideration:** written permission was sought for and obtained from the Institutional Review Board of the SDA Hospital Jengre before proceeding with the study.

## Results

A total of 168 PLHIV participated in this study with more females (63.7%) than the males (36.3%), age ranging from 20 to 66 years with the average age of 39.35 ± 8.32 years. More than half (86.9%) of the respondents had 1-4 children. About two-third (67.9%) of the respondents had been on ARV drugs for more than 5 years prior to the study and greater than half (58.3%) of the respondents had partners with concordant HIV status. Most (72.6%) of the respondents considered their health status to be very good (Table 1). Majority (81.5%) of the respondents were sexually active and most (63.1%) of them were involved in risky sexual behaviours. More than half (63.5%) of the respondents did not use any form of modern contraceptive to prevent pregnancy. A small proportion (3.6%) of the respondents used substance before having sex. In addition, most (74.5%) of the respondents did not use condom during their last sex even though some of them had multiple sexual partners (Table 2).

**Table 1:** sociodemographic and medical-related characteristics

Variables	Frequency	Percentage (%)
**Sex**		
Male	61	36.3
Female	107	63.7
**Age group (years)**		
≤34	56	33.3
35 - 44	77	45.8
≤45	35	20.8
**Educational status**		
No formal education	59	35.1
Primary	43	25.6
Secondary	44	26.2
Tertiary	22	13.1
**Marital status**		
Married	120	71.4
Single	10	6.0
Widowed	14	8.3
Divorced	17	10.1
Separated	7	4.2
**Duration of marriage (years)**		
≤4	25	15.8
5 - 9	27	17.1
≥10	106	67.1
**Number of children**		
0	22	13.1
1-3	93	55.4
≥4	53	31.5
**Duration since knowing HIV status (years)**		
≤4	44	26.2
≥5	124	73.8
**Duration on ARVs**		
≤4	54	32.1
≥5	114	67.9
**HIV disclosure**		
Disclosed	158	94.0
Not disclosed	10	6.0
**Partner's HIV status**		
Positive	98	58.3
Negative	45	26.8
Unknown	25	14.9
**Perceived current health status**		
Very good	122	72.6
Good	42	25.0
Fair	4	2.4
**Current viral load**		
Suppressed	144	85.7
Not suppressed	24	14.3

**Table 2:** sexuality and fertility desire of the respondents

Variables	Frequency	%
**Sexuality**		
Non-risky sexual behaviour	62	36.9
Risky sexual behaviour	106	63.1
**Had sex in the last 12 months**		
No	31	18.5
Yes	17	81.5
**Use of condom during last sex**		
No	102	74.5
Yes	35	25.5
**Number of sexual partners in the last 12 months**		
Single	118	86.1
Multiple	19	13.9
**Use of any form of contraceptive**		
No	87	63.5
Yes	50	36.5
**Use of substance before sex**		
No	132	96.4
Yes	5	3.6
**Fertility desire**		
Not desired	60	35.7
Desired	108	64.3
**Fertility desire by sex**		
Females	74	68.5
males	34	31.5
**Number of children desired**		
≤2	63	58.3
3 - 4	36	33.3
≥5	9	8.3
**Reasons why respondents had fertility desire**		
Due to family pressure	9	8.3
Due to social pressure	1	0.9
To replace myself	26	23.9
Yet to achieve desired family size	59	54.1
In a new relationship	17	15.6
Wants a male child	4	3.7
Wants a female child	2	1.8
To get support from them	4	3.7
**ART increased fertility desire**		
No	79	47.1
Yes	99	52.9
**Discussed fertility desire with healthcare provider**		
No	166	98.2
Yes	2	1.2
**Know any safe method of conception**		
No	168	100
Yes	0	0

About two-third (64.3%) of the respondents desired to have a child or more children in the future with more than half of them yet to achieve their desired family size. Use of ART increased the fertility desire in more than half of the respondents. Almost all (98.2%) the respondents have never discussed their fertility desires with their healthcare providers and none of the respondents had an idea on any safe method of conception aimed at reducing the risk of HIV transmission (Table 2). Concerning knowledge on MTCT of HIV, majority (62.5%) of the respondents had heard about MTCT of HIV. Knowledge on when MTCT of HIV can occur was poor as only 7.7%, 28.0% and 29.8% knew that it could occur during pregnancy, labour and breastfeeding respectively. Knowledge on whether MTCT could be prevented was also poor as only 28.0% of the respondents who had heard about MTCT knew that it could be prevented however 91.5% of this knew correctly how MTCT of HIV could be prevented. Overall knowledge on MTCT of HIV was poor with only 28.6% of the respondents having good knowledge (Table 3).

**Table 3:** knowledge of MTCT and the relationship with sexuality and fertility desire

Knowledge on MTCT						
**Variable**			**Frequency**		**%**	
**Heard of MTCT**						
No			63		37.5	
Yes			105		62.5	
**Knowledge on when MTCT of HIV can occur**						
**Pregnancy**						
No			155		92.3	
Yes			13		7.7	
**Labour**						
No			121		72.0	
Yes			47		28.0	
**Breastfeeding**						
No			118		70.2	
Yes			50		29.8	
**Knowledge that MTCT can be prevented**						
No			121		72.0	
Yes			47		28.0	
**Knowledge on how MTCT can be prevented**						
Incorrect			4		8.5	
Correct			43		91.5	
Over all knowledge						
Poor			120		71.4	
Good			48		28.6	
**Relationship between knowledge on MTCT and sexuality and fertility desire**						
**Variables**						
**Sexuality**						
	**Risky**		**Non-risky**	**Total**	**X2**	**P-value**
**Heard of MTCT**						
No	39 (61.9)		24 (938.1)	63	0.1767	0.6743
Yes	68 (64.8)		37 (35.2)	105		
**Knowledge on MTCT**						
Poor	73 (60.8)		47 (39.2)	120	0.5729	0.4491
Good	32 (66.7)		16 (33.3)			
**Fertility desire**						
	**No desire**		**Desire**	**Total**	**X2**	**P-value**
**Heard of MTCT**						
No	37 (58.7)		26 (41.3)	63	0.3878	0.5335
Yes	67 (64.2)		38 (35.8)	105		
**Knowledge on MTCT**						
Poor	76 (63.3)		44 (36.7)	120	0.0373	0.8468
Good	30 (62.5)		18 (37.5)	48		
						

Among the 105 respondents who were aware of MTCT of HIV 67 of them were involved in risky sexual behaviour, while 38 of those who were not aware of MTCT were also involved in risky sexual behaviour. Also 60.8% and 66.7% of the respondents who had poor and good knowledge of MTCT respectively were involved in risky sexual behaviours also. This study did not find any statistically significant relationship between either the awareness of MTCT or knowledge on MTCT with sexuality of the respondents even though awareness was found among majority of the respondents (Table 3).

Thirty-seven (58.7%) of the respondents who were not aware of MTCT of HIV and 67 (64.2%) of those who were aware of MTCT had fertility desires. In addition, 76 (63.3%) and 30 (62.5%) of the respondents with poor knowledge and good knowledge of MTCT respectively also had fertility desires. This study did not find a statistically significant relationship between either awareness or knowledge of MTCT with the fertility desires of the respondents even though majority of the respondents were aware of MTCT and also majority had poor knowledge on MTCT of HIV (Table 3). Only 2 (3.3%) of the respondents without fertility desires stated that they did not want to infect their baby as reason for no fertility desire.

## Discussion

Majority of the respondents had disclosed their HIV status to their partners. Some of the respondents were in an HIV sero-discordant relationship while few did not know the HIV status of their partners. These findings were similar to the results of studies done in Ethiopia and Tanzania [11,22]. The findings on disclosure of HIV status in this study is however, higher than that observed in Togo [23]. This may be as a result of the repeated counselling for PLHIV on their clinical visits to the hospital on the need for disclosure. Been in sero-discordant and unknown HIV status relationship increases the risk of HIV transmission.

This study found that majority of the respondents were sexually active and this was closely related to findings in Ethiopia [11], but was however higher in Tanzania, Togo, Denmark and Finland, Brazil, Democratic Republic of Congo, South Africa and Nigeria [22-28]. This may be due to the desire to have more children in the Northern part of Nigeria as a result of the dominant religion and also the practice of marrying more than one wife. This high rate of sexual activity found in this study is a concern since this can increase the risk of HIV transmission in sero-discordant partners and also re-infection of different strains of HIV for sero-concordant partners.

Condom use during last sex was very low as only a quarter of those who had sexual intercourse reported using condom. Low rates of condom use were also reported in Tanzania, Democratic Republic of Congo, Nigeria and South Africa [22,26,28,29]. Condom use was however, higher in studies in Ethiopia, Uganda, Brazil, Vietnam, Cameroun and Asia Pacific Region [11,15,25,30-32]. This low rate of condom use in this study may be due to the fact that most of the respondents were in sero-concordant relationship and may not had considered themselves at risk of re-infection but this however is a concern for partners who are in a sero-discordant relationship since they are at high risk of HIV infection. A very small proportion of the respondents used substance before sex and this was consistent with findings in Brazil and Togo [23,25].

Furthermore, having multiple sexual partners was revealed by this study and this was consistent with findings in Ethiopia, Togo, South Africa, Vietnam and Cameroun [11,23,27,30,31]. Also, close to two-third of the respondents did not use any form of contraceptive to prevent pregnancy and this was corroborated by result in Democratic Republic of Congo [26]. This is a concern as it increases risk of vertical transmission and also horizontal transmission of HIV in sero-discordant relationships.

A little below two-third of the respondents in this study had fertility desires and this was found more among the females. This finding was consistent with reports in similar studies in Democratic Republic of Congo, Canada and Jamaica [26,33,34]. Respondents in other studies done in Addis Ababa, Eastern Ethiopia, Uganda, Tanzania, Brazil and Kenya were however, found to have lower fertility desires [2,11,12,15,25,35]. The difference with the findings of these studies may be due to the fact that fertility rate in Northern Nigeria is high [36].

Concerning awareness, most of the respondents had heard of MTCT of HIV. Knowledge on when MTCT of HIV can occur was very poor as not up to a quarter of the respondents knew that MTCT could occur during pregnancy, while a little above a quarter each among the respondents knew that it could occur during labour and delivery respectively. Knowledge on whether MTCT could be prevented was also poor as only about a quarter of the respondents knew that MTCT could be prevented and majority of these knew correctly how MTCT of HIV could be prevented. Overall knowledge on MTCT of HIV was poor with just about a quarter of the respondents having good knowledge. Similar study in Ethiopia also revealed that awareness of MTCT was good but knowledge was poor [12]. In South Africa, West Ethiopia and Northwest Ethiopia [29,37,38] majority of the respondents were also aware of MTCT more than that of this study, but knowledge was also poor even though better than the findings of this study. This could be due to the fact that those studies were conducted among women attending ante-natal clinic (ANC) while our study included both men and non-pregnant women who most probably may never have had opportunity of receiving any education on MTCT since it is usually done in ANC settings.

This study did not show any association between either the awareness of MTCT or knowledge on MTCT with sexuality of the respondents even though awareness was good among majority of the respondents. This may be due to the fact that majority of the respondents were married and also to partners who are also living with HIV and therefore did not see any reason to adopt a non-risky sexual behaviour. It could also be due to the fact that all the respondents are all on ART and majority of them considered their health status as very good. This practice however, may increase the risk of MTCT considering the fact that majority of them did not use any form of contraceptive to prevent pregnancy. Also, no association was found between either awareness or knowledge of MTCT with the fertility desire even though majority of the respondents were aware of MTCT but with poor knowledge. This finding was consistent with study in Ethiopia [39]. Only a very small proportion of the respondents without fertility desires stated that they did not want to infect their baby as a reason. This high fertility desires found in this study even with awareness of MTCT of HIV may also be due to their poor knowledge. Again, this study included men and single women who constituted more than one-third of the respondents and may not have knowledge of MTCT of HIV since it is not usual in the setting of this study for men to attend ANC with their partners.

## Conclusion

Although this study showed high awareness and poor knowledge of MTCT of HIV, this however, did not affect the sexuality and fertility desires. Therefore, we recommend that information on MTCT of HIV be provided to PLHIV including men and single females by healthcare workers during routine clinic visits and not be limited to ANC in order to enable PLHIV make good decision about their sexuality and fertility desire to reduce HIV transmission risk.

**Limitations of study:** 1) sexual behaviour and practices are considered sensitive and personal issues in the Nigerian environment and so there could be some bias in the reported sexual practices. In order to control this, respondents were re-ensured of confidentiality on all information they provided for this study. Also, privacy was also ensured during interview session for each participant by attending to one participant at a time; 2) reported findings are limited by the validity of self-report and by possible recall bias. In order to reduce this, questionnaire was pre-tested on persons living with HIV who had similar characteristics with study participants in a different health facility to ensure quality of questionnaire. In addition, the research assistants who were involved in administering questionnaire were trained on the art of interview. Participants were also informed and re-assured about their anonymity as their names were not asked for in the course of the interview.

### What is known about this topic

- PLHIV have fertility desire;
- PLHIV have risky sexual behaviour.

### What this study adds

- Knowledge on MTCT does not affect sexuality in PLHIV;
- Knowledge on MTCT does not affect fertility desires in PLHIV.
